# Effects of the Decrease in Blood Lead Levels on Renal and Neurological Functions Among Occupationally Exposed and Unexposed Populations of South India: A Cohort Study

**DOI:** 10.7759/cureus.54432

**Published:** 2024-02-18

**Authors:** Arti Gupta, Mukesh Tripathi, Bari Siddiqui MA, Desai V Sripad, Vamsikrishna Reddy K, Desu Rama Mohan, Prudhvinath A Reddy, Rakesh Upparakadiyala

**Affiliations:** 1 Department of Community and Family Medicine, All India Institute of Medical Sciences, Mangalagiri, IND; 2 Department of Anesthesiology, All India Institute of Medical Sciences, Mangalagiri, IND; 3 Department of Biochemistry, All India Institute of Medical Sciences, Mangalagiri, IND; 4 Department of Hospital Administration, All India Institute of Medical Sciences, Mangalagiri, IND; 5 Department of Radiodiagnosis, All India Institute of Medical Sciences, Mangalagiri, IND; 6 Department of General Medicine, All India Institute of Medical Sciences, Mangalagiri, IND

**Keywords:** anemia, neurological, renal, occupation, lead

## Abstract

Introduction: Exposure to lead in excess of the permissible limit is a known risk factor leading to preventable morbidity. The present study aimed to assess whether there is a change in the neurological and renal parameters among adults with blood lead levels (BLLs) higher than recommended at baseline and after prevention among differently exposed adults.

Methodology: In the Guntur District of Andhra Pradesh, India, a cohort study was carried out in 2022-2023 among 180 adult males and females aged 20 to 60 years in three groups: direct occupational exposure, indirect occupational exposure, and no occupational exposure. If the blood levels were more than or equal to 5 mcg/dL, the participant's detailed neurological examination was done at baseline and end of follow-up. During the six-month follow-up period, health education on lead awareness was given monthly. BLLs were estimated using graphite furnace atomic absorption spectrometry (GFAAS). Serum creatinine was estimated using Jaffe's modified method. On neurological examination, reflexes, power, and sensation were assessed. The vibration perception threshold was determined using a biothesiometer. A p-value less than 0.05 was considered to be statistically significant.

Results: Among the 180 participants, the mean BLLs at baseline were 7.15±3.06 mcg/dL. The findings revealed a statistically significant decrease in mean BLLs at baseline to end of six-month follow-up. Despite this improvement, participants with BLLs ≥5 mcg/dL still accounted for a considerable proportion, albeit reduced, particularly in Groups 1 and 2. There were no statistically significant changes observed in the proportions of participants with abnormal serum creatinine, anemia, or abnormal neurological parameters.

Conclusion: These results suggest that while prevention activities may effectively reduce overall BLLs, there might be challenges in completely mitigating the impact on certain health parameters, such as renal and neurological functions.

## Introduction

The Centers for Disease Control and Prevention (CDC) updated the blood lead reference value from 5 mcg/dL to 3.5 mcg/dL in October 2021 [1]. In India, there are no specific national regulations regarding blood lead levels (BLLs) in factory workers and the general population. However, the Occupational Safety and Health Administration (OSHA) in the United States has established regulations for lead exposure. However, OSHA's general industry and construction lead standards include a medical removal protection provision for workers whose BLLs reach or exceed 50 mcg/dL (construction) or 60 mcg/dL (general industry) [2].

The US OSHA standard for lead exposure is followed by the Factory Act in India, allowing people to continue working in a lead-exposed environment with BLLs up to 40 mcg/dL [3]. However, studies have documented that the neurotoxic effects of lead in workers can be induced at BLLs below 18 mcg/dL [4]. Many longitudinal studies have provided evidence that cumulative lead doses cause cognitive dysfunction. The majority of these studies are among children, pregnant women, and occupationally exposed workers [4-6]. Once lead is absorbed into the bloodstream, some of it is filtered out and excreted, but the rest gets distributed to the liver, brain, kidneys, and bones. Lead causes anemia in adults by impairing the formation of oxygen-carrying molecules, beginning at exposures of around 40 mcg/dL [7].

We hypothesize that the neurological and renal manifestations are present even after BLLs return to normal. The OSHA acceptable criteria for considering lead toxicity in the Indian population is much higher, leading to an irreversible neurological deficit that is preventable. This study aimed to assess whether there is a change in the neurological and renal parameters among adults with BLLs higher than recommended at baseline and after prevention among differently exposed adults.

## Materials and methods

In the Guntur District of Andhra Pradesh, India, a cohort study was carried out in 2022-2023 among adult males and females aged 20 to 60 years in an area around 30 km surrounding All India Institute of Medical Sciences, Mangalagiri. Three groups of people were recruited to participate in the study: Group 1: direct occupational exposure (lead smelters, smolders, painters, construction workers, demolition workers, and gas station attendants); Group 2: indirect air pollution (traffic police, police, truck, bus, auto, and petrol bunk workers); and Group 3: not directly involved in the lead exposed occupation (indoor officer workers, teachers, primary healthcare providers, and housewives). Participants were eligible to participate if they were residents of the area for at least the last six months and members of Group 3, Group 1, and Group 2 who had been employed in the same profession for at least six months. Those who declined consent, had diabetes mellitus, hypertension, or surgery, or exhibited signs suggestive of a serious condition were excluded. The study was approved by the Ethics Committee of All India Institute of Medical Sciences Mangalagiri (approval number: AIIMS/MG/IEC/2022-23/13, approval date: 23-03-2022).

Using a t-test to compare the means of continuous variables, the sample size was determined using the calculation:

n= {(σ_1_^2^ + σ_2_^2^) * [Z_1-@/2 _+ Z _1- β_]^2^}/(M1-M2)^2^

where σ_1 _is the SD of the outcome variable in Group 1, σ_2 _is the SD of the outcome variable in Group 2, and M1-M2 is the mean difference to be detected.

Group 1 consists of laborers who handle raw materials in a battery industry located in Nellore, Andhra Pradesh. Their BLLs (mcg/dL) was 26.2±2.142 in 2016-2017 [3]. Group 2 comprises lead-exposed healthy school teachers who do not work in the public or government sector of Jodhpur and have BLLs (mcg/dL) of 6.89±9.5 [8]. For any two groups, a sample size of 60 was needed to detect a 3.0 mcg/dL difference in BLLs, with 80% power and 95% confidence intervals [9]. A total of 180 individuals were studied.

Information about the study was shared with communities via field health workers, schools, Anganwadi, and social media. They were to be a liaison participant for us in their routine field visit and brief us about the study. Any eligible participants in each of the three groups in the communities with the willingness to participate in the study were enrolled. Independent teams of investigators for coding and assessing exposure to lead, checklist-based clinical assessment, interviews, and blood sampling were formed. The investigator introduced himself/herself to the participant before the start of the interview. Individuals were given patient information sheets and were explained regarding the study, its objectives, procedure, and the rights of the participants. If the individuals agreed to participate in the study after going through the information sheet, then informed written consent was obtained from them. A unique code was given to the participant. The participants were interviewed according to the interview schedule. A blood sample for lead, hemoglobin, and serum creatinine estimation was done after the interview. Clinicians, laboratory technicians, and data analysts were blinded. The blood samples were transported to the laboratory, maintaining the cold chain.

If the BLL is <5 mcg/dL, the participant was retested for BLLs after six months. If the blood levels were ≥5 mcg/dL, the participants had a detailed neurological examination. They were retested for BLLs and neurological examination after six months. A structured data collection instrument comprising information about socio-demographic details, including age, smoking status, alcohol ingestion, and clinical details, was developed. This was pretested, suitably modified, and then implemented.

**Figure 1:**
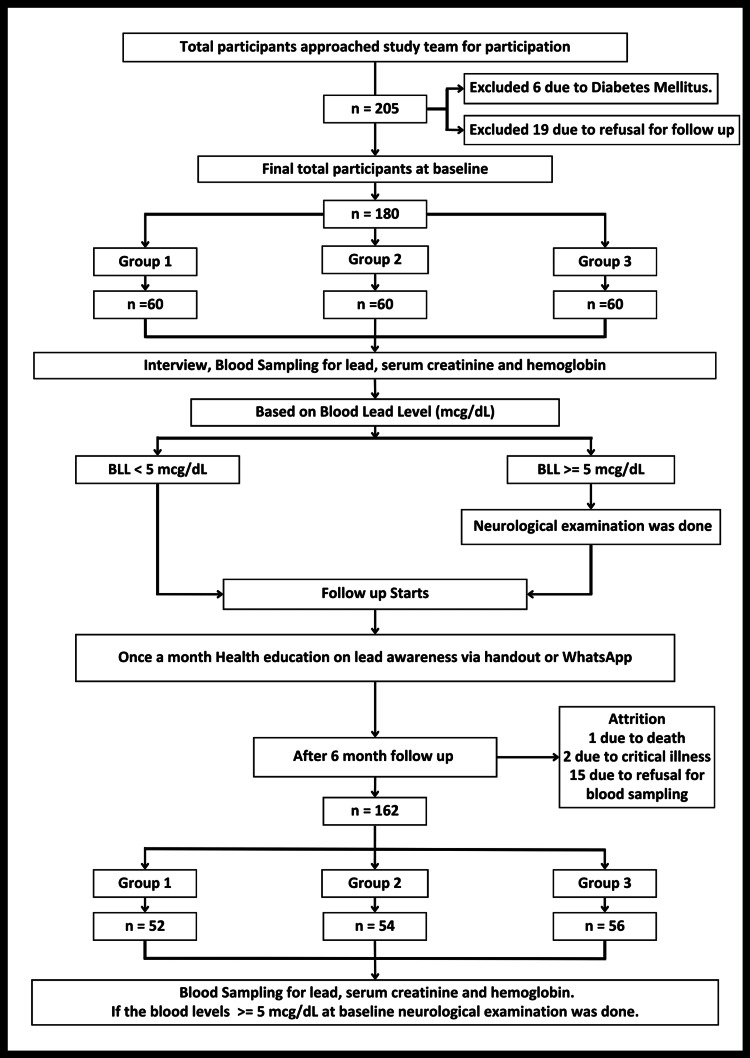
Flow diagram of study participants BLL: blood lead level

BLLs were estimated using a graphite furnace atomic absorption spectrophotometer (GFAAS) [10]. The method is validated, has a good detection limit (<1-2 mcg/dL), requires a small sample size, has a multielement capacity, and has very few interferences. Serum creatinine was estimated on a Beckman Coulter 700 AU (Beckman Coulter, Inc., CA, USA) using Jaffe’s modified method [11]. The normal range for serum creatinine levels was considered to be 0.74 to 1.35 mg/dL for adult males and 0.59 to 1.04 mg/dL for adult females. Hemoglobin was tested using the analyzer. Anemia was defined as hemoglobin <12 gm/dL among adult females and <13 gm/dL among adult males. On neurological examination, reflexes, power, and sensation were assessed. The vibration perception threshold (VPT) was determined using biothesiometry. Those with an average VPT of ≥15 V were considered abnormal. During the six-month follow-up period, health education on lead awareness was given every month through handouts or WhatsApp in a similar pattern in all three groups (Figure 1).

Data were entered into Microsoft Excel (Microsoft Corporation, WA, USA) and analyzed using SPSS Statistics version 28.0 (IBM Corp. Released 2021. IBM SPSS Statistics for Windows, Version 28.0. Armonk, NY: IBM Corp.). Lead exposures at baseline were categorized into three groups. The data were expressed as frequency, proportion, and mean ± SD. A one-way ANOVA was used to check the significance between various explanatory variables and BLLs among different groups. We defined statistical significance as p<0.05. The two-sample z-test for proportions was used to compare significant changes in proportions at baseline and end of follow-up surveys for abnormal serum creatinine, neuro-examination, and anemia. A paired t-test was used to compare significant changes in the mean of biochemical parameters at baseline and end of follow-up. A p-value less than 0.05 was considered to be statistically significant.

## Results

A total of 180 participants were studied at baseline. After the six-month follow-up period, 162 participants could be studied with an attrition rate of 10% due to death, illness, and withdrawal of participation. Of the total, 134 (74.4%) were males. Nearly two-thirds of participants were less than 40 years of age, 118 (65.6%), and educated to ≥10 standards, 123 (68.3%). The majority of the participants belonged to the upper middle 85 (47.2%) or middle 43 (23.9%) socioeconomic status. Males had significantly higher BLLs in all three groups compared to females (p<0.005). BLLs were also significantly higher among the three groups of low socioeconomic status and low alcohol use (Table 1).

**Table 1:** Comparison of BLLs among the three studied groups by various factors at baseline (n=180) BLLs: blood lead levels *p<0.05 was considered statistically significant

Domain	Category	N	%	Group 1		Group 2		Group 3		F statistics	p-value
				Mean	SD	Mean	SD	Mean	SD		
Age (in years)	<40	118	65.6	9.12	2.57	7.54	2.64	5.63	2.9	1.05	0.31
	≥40	62	34.4	7.98	2.07	7.05	3.53	5.95	3.15		
Gender	Female	46	25.6	0.00	0	5.10	0.42	5.71	3.09	16.26	<0.005*
	Male	134	74.4	8.50	2.36	7.41	3.04	5.48	2.42		
Education	<10 standard	57	31.7	8.47	2.62	7.65	2.06	4.55	1.3	2.08	0.15
	≥10 standard	123	68.3	8.51	2.13	7.18	3.42	5.82	3.05		
Migrant	Yes	43	23.89	8.78	2.68	6.80	2.5	6.90	4.13	0.01	0.91
	No	137	76.11	8.45	2.33	7.58	3.23	5.19	2.2		
Living with family	Yes	171	95.0	8.46	2.25	7.30	3.08	5.67	2.94	0.41	0.52
	No	9	5.0	9.02	4.03	7.96	1.73	5.05	2.33		
Socioeconomic status using BG prasad	Upper (INR 7889 and above)	45	25.00	6.90	1.44	6.18	2.63	5.64	2.81	4.83	0.003*
	Upper middle (INR 3944-7888)	85	47.22	8.36	2.25	8.00	3.59	5.26	2.33		
	Middle (INR 2367-3943)	43	23.89	9.35	2.17	8.36	1.94	6.72	4.39		
	Lower middle (INR 1183-2366)	7	3.89	7.22	3.39	7.50	0	5.80	0		
Smoking	Yes	32	17.78	9.35	2.59	7.39	2.58	0.00	0	4.64	0.03*
	No	148	82.22	8.26	2.26	7.31	3.23	5.65	2.91		
Smokeless tobacco use	Yes	7	3.89	8.92	2.98	0	0	0	0	2.53	0.11
	No	173	96.11	8.44	2.29	7.34	3.02	5.65	2.91		
Alcohol use	Yes	49	27.22	9.08	2.4	8.15	3.45	0	0	16.13	<0.001*
	No	131	72.78	8.16	2.29	6.67	2.48	5.65	2.91		
Area of living	Rural	75	41.6	8.61	2.39	7.88	2.69	5.18	2.65	0.93	0.39
	Urban	47	26.11	7.40	4.57	7.07	3.41	5.53	1.98		
	Peri-urban	58	32.22	8.50	2.14	6.60	1.88	6.25	3.58		
Drinking water source	RO	25	13.89	9.20		5.48	2.42	5.33	3.02	3.72	0.01*
	Canned water	91	50.56	8.39	2.41	7.63	3.08	5.97	2.56		
	Municipality	50	27.78	8.67	1.58	7.62	3.21	6.02	3.71		
	Others	14	7.78	8.66	5.7	7.52	2.28	4.57	1.68		
Total				8.50	2.36	7.34	3.02	5.73	2.92	15.91	<0.001*

There was a statistically significant decrease in mean BLLs at baseline (7.15±3.06 mcg/dL) and end of six-month follow-up (5.89±3.35 mcg/dL) with p<0.001. There was also a statistically significant decrease in hemoglobin at baseline (13.89±2.18 g/dL) and end of six-month follow-up (13.62±2.17 g/dL) with p<0.001 (Table 2).

**Table 2:** Summary of biochemical parameters at baseline and end of six-month follow-up survey *p<0.05 was considered statistically significant

Biochemical parameter	Baseline (n=162)		After six-month follow-up (n=162)		t	p-value
	Mean	SD	Mean	SD		
Blood lead (mcg/dL)	7.15	3.06	5.89	3.35	6.50	<0.001*
Serum creatinine	0.75	0.21	1.60	7.34	-1.46	0.15
Haemoglobin (gm/dL)	13.89	2.13	13.62	2.17	3.19	<0.001*

The proportion of BLLs ≥5 mcg/dL significantly decreased from 72.2% to 50.6% among participants (n=162) at baseline and end of follow-up surveys, respectively. On further stratification of participants into occupation groups, the proportion of BLLs ≥5 mcg/dL among Group 1 significantly dropped from 92.3% (95%CI: 85.1 to 99.5) to 67.3% (95%CI: 54.5 to 80.0) at baseline and the end of follow-up surveys among participants (p=0.001). In addition, the proportion of BLLs ≥5 mcg/dL among Group 2 also significantly dropped from 75.9% (95%CI: 64.4 to 87.3) to 53.7% (95%CI: 40.4 to 67.0) at baseline and end of follow-up surveys among participants (p=0.015) (Table 3).

**Table 3:** Comparison of proportion of BLLs more than or equal to 5 mcg/dL at baseline and end of six-month follow-up survey BLLs: blood lead levels *p<0.05 was considered statistically significant

Domain		Participants with blood levels more than or equal to 5 mcg/dL						z-test	p-value
	Total	Baseline survey			End survey				
	n	n	%	(95% CI)	n	%	(95% CI)		
Step 1: number of participants excluding attrition	162	117	72.2	65.1-78.9	82	50.6	42.3-57.7	4.1	<0.0001*
Step 2: stratifying participants for occupation									
Group 1	52	48	92.3	85.1-99.5	35	67.3	54.5-80.0	3.2	0.0015*
Group 2	54	41	75.9	64.4-87.3	29	53.7	40.4-67.0	2.4	0.0157*
Group 3	56	27	48.2	35.1-61.2	18	32.1	19.9-44.3	1.7	0.0822

There was no statistically significant change using the z-test for proportion among participants with abnormal serum creatinine at baseline and end of follow-up surveys and with stratification by occupation and BLLs (Table 4).

**Table 4:** Comparison of proportion of abnormal serum creatinine at baseline and end of six-month follow-up survey BLL: blood lead level *p<0.05 was considered statistically significant

Domain	Participants with abnormal serum creatinine							z-test	p-value
	Total	Baseline survey			End survey				
	n	n	%	(95% CI)	n	%	(95% CI)		
Step 1: participants excluding attrition	162	62	38.3	30.81-45.8	69	42.6	35-50.2	0.8	0.4304
Step 2: stratifying participants with respect to occupation									
Group 1	52	19	36.5	23.41-59.5	20	38.5	25.2-51.7	0.2	0.8332
Group 2	54	18	33.3	20.7-45.9	17	31.5	19.1-43.9	0.2	0.8416
Group 3	56	25	44.6	31.5-57.6	32	57.1	44.1-70.0	1.3	0.1858
Step 3: stratifying participants with respect to BLLs									
Group 1: BLL <5 mcg/dL	4	2	50	01.00-99.0	2	50	01.0-99.0	0	1
Group 1: BLL ≥5 mcg/dL	48	17	35.4	21.9-49.0	18	37.5	23.8-51.2	0.2	0.8307
Group 2: BLL <5 mcg/dL	13	2	15.4	-04.22-35.0	4	30.8	05.7-55.9	0.9	0.3516
Group 2: BLL ≥5 mcg/dL	41	16	39	24.0-54.0	13	31.7	17.4-46.0	0.7	0.4893
Group 3: BLL <5 mcg/dL	29	11	37.9	20.2-55.5	18	62.1	44.4-79.7	1.8	0.0653
Group 3: BLL ≥5 mcg/dL	27	14	51.9	33.0-70.7	14	51.9	33.0-70.7	0	1

There was no statistically significant change using the z-test for the proportion among participants with anemia at baseline and end of follow-up surveys, with stratification by occupation and BLLs (Table 5).

**Table 5:** Comparison of proportion of anemia at baseline and end of six-month follow-up survey BLL: blood lead level

Domain	Participants with anemia							z-test	p-value
	Total	Baseline survey			End survey				
	n	N	%	(95% CI)	n	%	(95% CI)		
Step 1: participants excluding attrition	162	135	83.3	77.56-89.0	128	79	72.7-85.2	1	0.3224
Step 2: stratifying participants for occupation									
Group 1	52	51	98.1	94.3-101.8	50	96.2	91.0-101.4	0.6	0.5604
Group 2	54	51	94.4	88.2-100.5	51	94.4	88.2-100.5	0	1
Group 3	56	33	58.9	46.0-71.7	27	48.2		1.1	0.2563
Step 3: stratifying participants for BLLs									
Group 1: BLL <5 mcg/dL	4	4	100	-19.4-39.4	4	100	-19.4-39.4	0	1
Group 1: BLL ≥5 mcg/dL	48	47	97.9	93.8-101.9	46	95.8	90.1-101.4	0.6	0.5559
Group 2: BLL <5 mcg/dL	13	12	92.3	77.8-106.7	12	92.3	77.8-106.7	0	1
Group 2: BLL ≥5 mcg/dL	41	39	95.1	88.4-101.7	39	95.1	88.4-101.7	0	1
Group 3: BLL <5 mcg/dL	29	17	58.6	40.6-76.5	14	48.3	30.1-66.4	0.8	0.4317
Group 3: BLL ≥5 mcg/dL	27	16	59.3	40.7-77.8	13	48.1	29.2-67.0	0.8	0.4092

There was no statistically significant change using the z-test for proportion among participants with BLLs ≥5 mcg/dL with abnormal VPT, abnormal reflexes, and abnormal muscle power at baseline and end of follow-up surveys and with stratification by occupation (Table 6).

**Table 6:** Comparison of proportion of abnormal neurology examination at baseline and end of six-month follow-up survey

Participants with VPT ≥15 V									
Domain	Total	Baseline survey			End survey			z-test	p-value
	n	n	%	(95% CI)	n	%	(95% CI)		
Step 1: participants excluding attrition	116	25	21.6	14.11-29.0	15	12.9	6.8-19.0	1.8	0.0795
Step 2: stratifying participants with respect to occupation									
Group 1	48	10	20.8	09.3-32.3	8	16.7	6.15-27.2	0.5	0.6068
Group 2	41	11	26.8	13.2-40.3	6	14.6	3.7-25.4	1.4	0.1728
Group 3	27	4	14.8	1.4-28.1	1	3.7	18.7-55.2	1.9	0.0626
Participants with any abnormal reflex									
Step 1: participants excluding attrition	116	24	20.7	13.3-28.0	20	17.2	10.3-24.0	0.7	0.4964
Group 1	48	9	18.8	7.7-29.8	5	10.4	1.7-19.0	1.2	0.2439
Group 2	41	8	19.5	7.7-31.6	7	17.1	5.5-28.6	0.3	0.7787
Group 3	27	7	29.5	12.3-46.7	8	29.6	12.3-46.8	0	0.9936
Participants with power 3 or less in any muscle									
Step 1: participants excluding attrition	116	3	2.6	18.0-33.9	10	8.6	79.6-92.3	9.2	0
Group 1	48	1	21.1	95.6-32.6	7	14.6	4.6-24.5	0.8	0.4057
Group 2	41	2	4.9	33.7-64.3	2	4.9	33.7-64.3	0	1
Group 3	27	0	0	0	1	3.7	18.7-55.2	3.5	0.0005

## Discussion

The present study is a first-of-its-kind community-based cohort study in India that studied whether any changes occur in neurological and renal parameters in adults with elevated BLLs at baseline and after prevention for six months. The study's assessment of the overall mean BLLs at baseline, averaging 7.15±3.06 mcg/dL, reveals a concerning elevation compared to the recently updated blood lead reference value by the CDC [1]. The stratification of the participants into three distinct groups based on occupational exposure provides valuable insights into the varying degrees of BLLs within different occupational categories: Group 1, consisting of individuals with direct occupational exposure to lead, exhibited higher BLLs compared to both Group 2 and Group 3. This observation aligns with the facts, as these professions involve direct contact with lead-containing materials and processes, resulting in an increased risk of lead exposure. Group 2 individuals vulnerable to indirect air pollution demonstrated higher BLLs compared to Group 3. This finding highlights the impact of environmental exposure, particularly through air pollution generated by vehicular activities. The elevated BLLs in Group 2 emphasize the importance of considering not only direct occupational exposure but also indirect environmental factors when assessing lead exposure risks. Group 3, consisting of individuals not directly involved in lead-associated occupations, exhibited comparatively lower BLLs but higher than permissible levels. This suggests that their occupations involve minimal direct exposure to lead-containing materials or processes. However, it's crucial to note that even within this group, individuals may still encounter environmental lead exposure, albeit to a lesser extent than the occupational groups in Groups 1 and 2.

The observed reduction in the proportion of individuals with BLLs ≥5 mcg/dL at baseline and end of follow-up surveys among the overall participant cohort is significant. Moreover, the study's findings demonstrate a significant reduction in the proportion of individuals with BLLs ≥5 mcg/dL over the study period, particularly within high-risk occupational groups. This underscores the importance of preventive activities and emphasizes the importance of targeted strategies to further reduce lead exposure.

However, the lack of statistically significant changes observed in various health parameters, including abnormal serum creatinine levels, anemia prevalence, and neurological assessments, between baseline and end of follow-up surveys indicates that even prevention activities during the study period did not have a discernible impact on these health outcomes.

Irreversible damage to kidney and neurological functions can occur as a consequence of exposure to elevated BLLs. Similarly, another study documented possible kidney dysfunction at BLLs of 5-9 mcg/dL and possible neurological dysfunction at BLLs ≥5 mcg/dL [12]. According to a prospective Swedish population-based cohort study in 2018, among 4,341 individuals aged 46 to 67 years, after controlling for known risk factors, individuals with high median lead concentrations had higher eGFR changes from baseline to follow-up compared with those in the lowest quartiles [13]. In 2010, a study from the USA with 769 adolescent participants aged 12 to 20 years found that as high as 10 mcg/dL BLLs were associated with a lower estimated glomerular filtration rate (eGFR) [14]. Recent large-scale, prospective studies suggest that BLLs below 10 mcg/dL significantly worsen neurological function [15].

What is particularly concerning is that even if BLLs are reduced to normal or lower levels, the adverse effects on kidney and neurological functions may persist, showcasing a lack of reversibility in the damage incurred. BLLs, even at concentrations below OSHA's cutoff guidelines [2], have the potential to cause irreversible damage, particularly to the kidneys and nervous system.

In the current study, it was observed that the mean BLLs exceeded the recommended limits across all three examined groups. It is plausible that occupational exposure is a contributing factor to the higher BLLs observed in this population. Lead poses a significant risk to the central nervous system, and its accumulation in the kidneys can result in kidney damage. There was no statistically significant change in the proportion of participants with abnormal serum creatinine, abnormal VPT, abnormal reflexes, abnormal muscle power, and anemia at baseline and end of follow-up surveys, despite a statistically significant decrease in the proportion of participants with BLLs ≥5 mcg/dL. While decreasing lead exposure is an essential step in preventing kidney and neural damage, it may not guarantee immediate or complete improvement in kidney and neurological function, and additional measures may be necessary for addressing kidney and neurological damage.

Nevertheless, the primary limitation of the study is that while the participants had abnormal serum creatinine, abnormal VPT, abnormal reflexes, abnormal muscle power, and anemia, establishing a direct causal relationship solely based on BLLs is challenging. Comprehensive clinical and biological investigations are necessary to rule out alternative causes for these findings. In addition, a limited follow-up time of six months and the self-selection of participants can further affect the outcome.

## Conclusions

The study's findings demonstrate there was no change in serum creatinine, hemoglobin, or neurological functions despite a significant reduction in the proportion of individuals with BLLs ≥5 mcg/dL over the study period. This reinforces the critical importance of reevaluating the blood lead cutoff under the Factory Act. Stringent preventive measures and ongoing health monitoring in populations at risk of lead exposure are the utmost needs.
